# Supplementary figures and images for: BCL-2 Expression in AML Patients over 65 Years: Impact on Outcomes across Different Therapeutic Strategies
Source: J Clin Med. 2021 Oct 30;10(21):5096. doi: 10.3390/jcm10215096 (PMC8585096; doi:10.3390/jcm10215096)

# BD FACSDiva 8.0.2

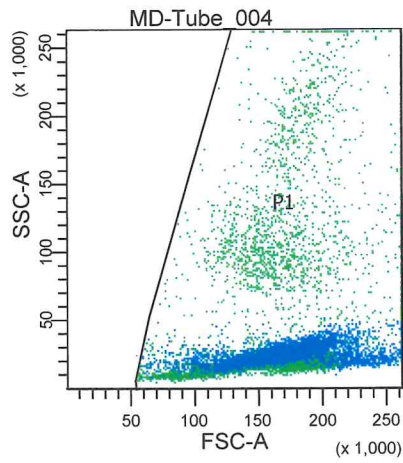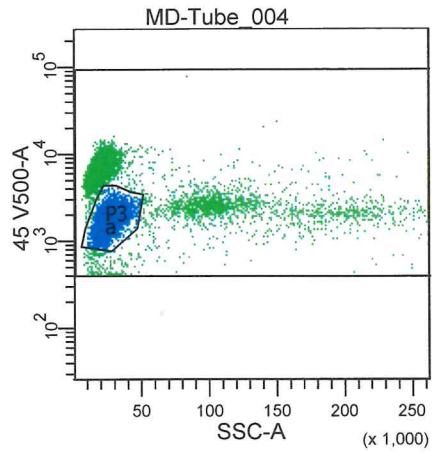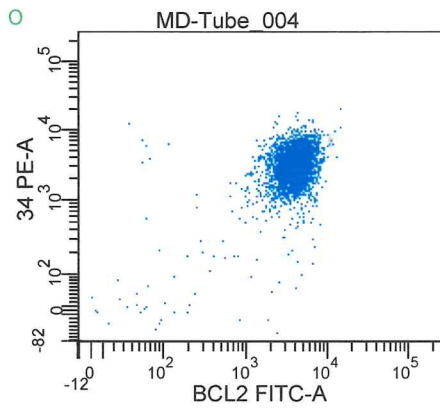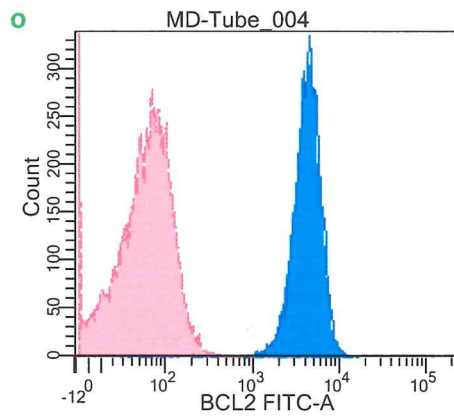

Supplement: Supplementary file 1 [file jcm-10-05096-s001.zip › jcm-1377651-supplementary.pdf]
